# How Machiavellianism, Psychopathy, and Narcissism Affect Sustainable Entrepreneurial Orientation: The Moderating Effect of Psychological Resilience

**DOI:** 10.3389/fpsyg.2019.00779

**Published:** 2019-04-12

**Authors:** Wenqing Wu, Hongxin Wang, Hsiu-Yu Lee, Yu-Ting Lin, Feng Guo

**Affiliations:** ^1^College of Management and Economics, Tianjin University, Tianjin, China; ^2^Department of Business Administration, Cheng Shiu University, Kaohsiung, Taiwan; ^3^Department of Food and Beverage Management, Cheng Shiu University, Kaohsiung, Taiwan

**Keywords:** Machiavellianism, psychopathy, narcissism, psychological resilience, sustainable entrepreneurial orientation

## Abstract

In order to solve increasingly serious environmental problems, sustainable entrepreneurship based on the concept of sustainable development has gradually attracted attention and discussion in the academic field. Moreover, it is of great significance to analyze the influence of personality on entrepreneurial orientation considering dark and bright personality traits. On the basis of existing research, we analyzed the relationships between the three elements of the dark triad (Machiavellianism, psychopathy, and narcissism), psychological resilience, and sustainable entrepreneurial orientation (SEO) through a questionnaire survey. Results involve data from 328 MBA students in Tianjin University of China. The results showed that Machiavellianism and psychopathy negatively affected SEO, and narcissism positively affected SEO; psychological resilience and SEO had a significant positive association; and psychological resilience had a moderating effect on the relationship between the three elements of the dark triad and SEO. Our research has reference value and its findings have important implications for entrepreneurship research and education.

## Introduction

With increasingly prominent environmental problems such as global climate change and water and soil pollution, academics have started to consider ways to address these problems (Chang and Park, 2018). As one of the breakthroughs to solve environmental problems, sustainable entrepreneurship, based on the concept of sustainable development, has attracted the attention of scholars (Chang and Park, 2018). It has been suggested that entrepreneurship should be examined from the perspective of sustainability (Kuckertz and Wagner, 2010; Stål and Bonnedahl, 2016), as scholars believe that in the process of entrepreneurship, start-ups can help reduce environmental pollution at the source by creating green and sustainable products/services. Moreover, achieving economic success is no longer the sole purpose of entrepreneurship (Kuckertz and Wagner, 2010). Sustainable entrepreneurship combines environmental protection with profit realization, which may not only help start-ups discover new entrepreneurial opportunities, but may also enhance their dual benefit: economic and social values. In particular, for developing countries with economies in transition, such as China, sustainable entrepreneurship can effectively transform the economic development model of “treatment after pollution,” which is of vital significance to the healthy economic development of such countries. From the macro view, sustainable entrepreneurship certainly has an increasingly relevant position and role. From the micro perspective, whether entrepreneurs have a sustainable entrepreneurial orientation (SEO) may be particularly important, as SEO can help translate sustainable entrepreneurship from theory into practice.

With the gradual deepening of research on SEO in recent years, scholars have begun to focus on the impact of individual personality traits on entrepreneurial orientation (Smith et al., 2017). However, existing studies only analyzed the influence of either bright or dark traits of personality on entrepreneurial orientation, and little attention has been paid to the interactions between dark and bright personality traits (Smith et al., 2017). Moreover, existing research incorporated the concept of sustainable development into entrepreneurial orientation and proposed SEO. These preliminary studies focused on the concept and connotations of SEO (Criado-Gomis et al., 2017; Chang and Park, 2018). Thus, few studies have analyzed the impact of personality traits on SEO.

Based on the deficiencies of existing research, our study aimed to analyze the respective effects of bright and dark personality traits on SEO. On the one hand, considering that sustainable entrepreneurship is a kind of social entrepreneurship, as a non-profit entrepreneurship, the possibility of failure is very high. Entrepreneurs can only succeed if they have a strong heart, that is, psychological resilience, which is the ability to cope with major changes, adversities, and risks (Duchek, 2018). Psychological resilience is an important quality for entrepreneurs (Korber and McNaughton, 2018) and plays a key role in the process of entrepreneurship (Duchek, 2018). It helps individuals to develop and mobilize resources they can use in the face of adverse circumstances, which is a vital growth strategy for entrepreneurs (Ayala and Manzano, 2014). Therefore, our study adopted psychological resilience as the representative of bright personality traits. On the other hand, the dark triad (i.e., Machiavellianism, psychopathy, and narcissism) has become a leading model for the study of dark personality traits in the psychological field (Adrian et al., 2013) and business management research (Smith et al., 2016). Therefore, our study adopted the dark triad as the representative of dark personality traits.

In addition, existing research focused on the impact of individual personality traits on entrepreneurial orientation at the organizational level (Kraus et al., 2018). However, potential entrepreneurs have not yet entered entrepreneurial organizations, which require an analysis of entrepreneurial orientation at the individual level. Therefore, SEO in this study was assessed at the individual level and refers to the behavioral tendency of potential entrepreneurs to achieve sustainable development and environmental protection through entrepreneurship, based on their concern for the environment. In summary, our study aimed to analyze the effects of the dark triad and psychological resilience on SEO at an individual level. At the same time, we aimed to analyze the moderating effect of psychological resilience on the dark triad and SEO, and thus explore the influence mechanism and path of personality traits on SEO more deeply.

Our study mainly makes the following three contributions to previous research. First, we adopted bright and dark personality traits as antecedent variables to analyze their respective impact on SEO. Second, our study aimed to clarify the moderating effect of psychological resilience on the dark triad and SEO through multiple regression analysis, responding to the appeal of Smith et al. (2017). Third, our study analyzed the impact of personality traits on SEO at the individual level, further enriching existing research on SEO and expanding the research framework, while helping to more fully understand SEO in individuals who have not yet entered entrepreneurial organizations.

### Theoretical Background and Hypothesis Development

#### The Dark Triad Traits and SEO

The three elements of the dark triad include Machiavellianism, psychopathy, and narcissism (Paulhus and Williams, 2002), which are mainly manifested in exploitative social strategies that promote personal goals (O’Boyle et al., 2012). The dark triad involves social malice with a tendency to self-improving behavior and reflects an unpopular interpersonal behavior style (Lee and Ashton, 2005). Its main characteristics are emotional coldness, exploitation, manipulativeness, and grand self-importance (Paulhus and Williams, 2002; Lee and Ashton, 2005). A large amount of evidence shows that the dark triad is related to negative behaviors and characteristics, such as dishonest behavior (Adrian et al., 2013). Moreover, the dark triad often leads potential entrepreneurs to neglect moral and social responsibility, and affects their perception of corporate social responsibility (Myung and Yun, 2017). In fact, corporate social responsibility is not only reflected in public welfare and charity, but also in the importance attached to the environment and its protection. In most cases, environmental problems are understood as social dilemmas with time conflicts, representing tensions between the satisfaction of short- and long-term interests (Milfont et al., 2012). For example, Huang et al. (2018) showed that the dark triad was negatively correlated with environmental attitudes and suggested that when individuals with the dark triad weigh short-term interests and future environmental interests, they tend to satisfy their short-term interests at the expense of future environmental interests, which is ultimately expressed as negative environmental attitudes.

In addition, Smith et al. (2017) pointed out that social cognitive theory and social exchange theory provide an excellent framework to study the results of harmful behaviors related to the dark triad. Social exchange theory poses that those who can provide us with the highest returns are most attractive, and individuals always try to obtain the greatest rewards for themselves from their social interactions. In order to obtain these returns, the corresponding price has to be paid (O’Boyle et al., 2012). Based on moral disengagement discussed in social cognitive theory (Bandura, 1999), individuals with the dark triad may be more likely to experience moral disengagement than those with other personalities. Combining social cognitive theory with social exchange theory, we proposed that individuals with the dark triad may have a more negative perception of society, which leads them to believe that social exchange based on the concept of sustainable development will achieve less benefits, while they have a desire to obtain higher returns in the short term. Therefore, individuals with the dark triad may be more likely to gain short-term benefits from social exchanges at the expense of the environment, rather than adopting a SEO.

##### Machiavellianism

Machiavellianism is characterized by self-interest, lack of empathy, and interpersonal manipulation (Furtner et al., 2011). These negative characteristics may lead individuals with Machiavellianism to ignore the importance of the environment, and even choose the former in the trade-off between short-term benefits and long-term development. Specifically, on the one hand, individuals with Machiavellianism may try to gain advantage by any necessary means, without considering morality (Myung and Yun, 2017). Individuals high in Machiavellianism have a strong demand for money and wealth (Zettler and Solga, 2013), a desire to control others and pursue status (Dahling et al., 2009), and are good at using opportunities to maximize their profits (Liu, 2008). They tend to adopt short-term strategies that provide immediate gratification and are closely related to living a “fast life” (Jonason and Tost, 2010; Hmieleski and Lerner, 2016). Thus, long-term environmental benefits may not be important to these individuals. On the other hand, individuals with Machiavellianism establish and achieve their goals by controlling others, emphasizing practicality and eagerness for power. They tend to use their organizations and other people as a means for them to achieve their goals, and are more likely to engage in anti-production work (Dahling et al., 2009) without regard for the importance and protection of the environment. That is to say, individuals with Machiavellianism are mostly self-concerned, only focus on their own goals, and rarely consider the consequences for those around them and the surrounding environment (Zettler et al., 2011). In short, individuals with Machiavellianism follow their own goals, engage in low-emotional interactions, and have a low ideological input (Zettler et al., 2011). They struggle only for personal goals and tend to ignore ethical and environmental issues. For example, Simmons et al. (2013) showed a negative correlation between Machiavellianism and perception of business ethics.

##### Psychopathy

Psychopathy is characterized by high impulsivity, thrill-seeking behavior, low empathy, lack of loyalty, and irresponsibility (Boddy et al., 2010). Psychopaths are often attracted to power, prestige, and control (Deutschman, 2007), which leads them to focus on short-term benefits, maximize their wealth and power by making short-term decisions (Boddy, 2006), and achieve short-term economic benefits even at the expense of the environment. It was reported that corporate psychopaths constitute the greatest threat to global business ethics (Marshall et al., 2014). Previous studies have argued that psychopaths have no conscience, and are not at all concerned with the consequences of their behavior on the environment, which implies a serious lack of social responsibility (Boddy, 2015). This conclusion is supported by a study by Boddy (2010), which found that the presence of psychopathic leaders leads to a lack of corporate social responsibility in an organization. Such organizations are unlikely to conduct their business in an environmentally friendly way or in a way that benefits the local community (Boddy, 2010). In addition, individuals high in psychopathy abhor social norms and are willing to oppose the status quo (Mathieu et al., 2013), and often lack empathy. Thus, they may be inclined to turn a blind eye to increasingly serious environmental problems and even do things that are not conducive to preserving the environment, which is exactly in line with the negative traits and behavior patterns of psychopaths.

##### Narcissism

The main characteristics of narcissism are domination, expressionism, and exploitation, as well as superiority and entitlement (Lee and Ashton, 2005). Narcissism, as a multifaceted dimension of personality, captures the extent to which an individual has an inflated self-consciousness and is constantly focused on himself (Chatterjee and Hambrick, 2007). Narcissists tend to overestimate their creativity, abilities, and wisdom (Myung and Yun, 2017). They believe that social exchanges based on the concept of sustainable development provide less benefits and take longer, and have a desire for high returns in the short term. Therefore, they are more likely to choose to gain some immediate benefits through short-term actions than to achieve long-term benefits through sustainable development. Specifically, on the one hand, narcissists exhibit anti-social, radical, and sadistic attitudes, which are detrimental to the promotion of sustainable development and entrepreneurship, as they lack compassion and show arrogant or contemptuous behavior or attitudes. Moreover, narcissists are often regarded as self-important and insensitive (Wales et al., 2013). They tend to be self-centered and constantly focus on themselves, without attending to social or environmental issues around them, and even show some anti-social behavior. On the other hand, the negative consequences of narcissism are well-documented in the existing literature (Resick et al., 2009). Narcissists are primarily concerned with behaviors focused on themselves, which often lead to power, shameless self-admiration, excessive arrogance, and resistance to external criticism (Resick et al., 2009). Therefore, they may be more concerned about personal development, ignoring the importance of those around them and environmental protection, which is not conducive to the adoption of SEO. Therefore, we proposed the following hypothesis:

**H1: (a) Machiavellianism, (b) psychopathy, and (c) narcissism are negatively associated with SEO.**

#### Psychological Resilience and SEO

Psychological resilience refers to the ability of individuals to successfully respond to major changes, adversities, or risks (Duchek, 2018). This study mainly analyzed resilience introduced from health and psychology research into the context of entrepreneurship (Bullough and Renko, 2013). Psychological resilience in the context of entrepreneurship refers to the ability to overcome high-impact entrepreneurial challenges and persist in the entrepreneurial process in the face of adverse situations and unexpected outcomes (Awotoye and Singh, 2017). The definition of psychological resilience has three key themes: adversity, positive adaptation, and risk/uncertainty, which are relevant in the entrepreneurship context. The concepts of risk and uncertainty are core in entrepreneurship because the entrepreneurial process involves significant risks (Awotoye and Singh, 2017). Therefore, we believe that psychological resilience is closely related to entrepreneurship. In fact, entrepreneurship under adverse conditions depends to a large extent on entrepreneurs’ awareness and resilience in the face of adversity (Bullough and Renko, 2013), as individuals who do business in complex situations often need to challenge the status quo and open up new avenues for success. Without resilience, individuals would not be able to engage in entrepreneurship or entrepreneurial behavior needed to pursue a new business (Bullough and Renko, 2013).

In addition, from the perspective of social cognitive theory, individuals with psychological resilience are more likely to identify with sustainable development, and their resilience and optimism make them more inclined to engage in sustainable entrepreneurship. At the same time, based on social exchange theory, in social exchange relationships (e.g., between individuals and the environment), individuals with psychological resilience might be more likely to agree with the concept of sustainable development because, in their view, seeking harmony with the environment, despite its costs, would provide them with more long-term benefits, such as good reputation for their enterprise, sustainable development, and so on. In short, psychological resilience has an important impact on entrepreneurship, and psychologically resilient individuals are more likely to adopt sustainable entrepreneurship and behavioral tendencies.

Through exploratory factor analysis, Ayala and Manzano (2014) found that hardiness, resourcefulness, and optimism are the three factors of entrepreneurs’ psychological resilience. First, hardiness involves a kind of self-control that prevents individuals from getting easily frustrated in the face of adverse circumstances, while being bold and striving to achieve their goals (Kobasa, 1979). Additionally, personal resilience has been found to be associated with a range of positive personal attitudes and behaviors (Sommer et al., 2016). By combining resilience resources with protective factors, individuals perform better and stay healthy under high pressure (Avey et al., 2011). Hardiness prevents individuals from being tempted by immediate short-term interests, and can lead them to firmly adhere to the concept of sustainability and thus achieve long-term environmental and other benefits.

Second, resourcefulness refers to the resources, abilities, and skills that individuals possess to control the various adverse conditions they must face. Resourcefulness means that individuals believe in their ability to control events and influence the outcome of situations (Powell and Baker, 2011). In real life, entrepreneurs usually exhibit many characteristics related to resourcefulness. For example, they tend to perform well in the face of ambiguity and change (Ayala and Manzano, 2014), consider a “terrible situation” as an opportunity (Bullough et al., 2014), persist in adversity (Holland and Shepherd, 2013), and ultimately act and achieve goals with wisdom and endurance. In fact, entrepreneurs’ resourcefulness is not entirely innate, and a large part of it is acquired through learning. Resilient entrepreneurs have a greater ability to self-renew through innovation over time and adapt to diverse and turbulent changes in the environment (Baardwijk and Reinmoeller, 2005). They can “stand up” again after failure and become stronger than before (Cannon and Edmondson, 2005). This kind of resourcefulness helps them to find business opportunities in adversity and learn from failures, so that they can better cope with environmental problems, discover entrepreneurial opportunities, and ultimately achieve sustainable entrepreneurship and long-term benefits.

Third, optimism refers to the ability of individuals to maintain a positive attitude in difficult situations (Schneider, 2001). Individuals can learn from mistakes and see them as opportunities rather than failures (Schneider, 2001). Studies have shown that optimism has a positive impact on entrepreneurship and helps entrepreneurs actively deal with various problems. Therefore, optimist individuals may be more proactive in dealing with environmental problems and find it easier to cultivate their own creativity. Optimism can encourage entrepreneurs to maintain entrepreneurial motivation and actively focus on environmental and social issues around them, thereby improving their SEO. In short, individuals high in psychological resilience can take positive actions in adversity, may not be discouraged by the challenges of environmental problems, and can learn to live with rejection. Compared with others, they have higher motivation and more positive attitude to solve environmental and social problems (Bullough et al., 2014). Therefore, we hypothesized that psychological resilience would play a positive role in promoting SEO as follows:

**H2: Psychological resilience is positively related to SEO.**

#### Moderating Effect of Psychological Resilience

According to the previous discussion, the dark triad and psychological resilience have different effects on SEO. Specifically, the three elements of the dark triad (Machiavellianism, psychopathy, and narcissism), though conceptually different, share some common negative characteristics (Kraus et al., 2017), for example, emotional coldness, exploitation, manipulativeness, and grand self-importance (Paulhus and Williams, 2002; Lee and Ashton, 2005). Based on the previous analysis, these personality traits may have a negative impact on SEO. In addition, in the entrepreneurial environment, resilience as a dynamic adaptation process is the ability of entrepreneurs to overcome a particularly difficult environment. In this process, entrepreneurs acquire knowledge, abilities, and skills through interaction with the environment, and rely on their own resources to face an uncertain future with a positive attitude (Windle et al., 2011; Ayala and Manzano, 2014). Similarly, based on the previous analysis, we believe that psychological resilience has a positive impact on SEO.

Simultaneously, when the level of individual psychological resilience is different, the influence of the dark triad on SEO may also be different. For individuals with dark triad, their ruthlessness, irresponsibility, and arrogant attitude would only make them pay more attention to personal development, while rarely consider the consequences for those around them and the surrounding environment (Huang et al., 2018). Furthermore, they would take a negative attitude toward sustainable entrepreneurship which is full of challenges and pursues long-term benefits. In particular, when individuals with dark triad have a high level of psychological resilience, this negative attitude toward sustainable entrepreneurship may be weakened. Because when individuals have higher levels of psychological resilience, their self-confidence to overcome difficulties and ability to cope with adversity would be correspondingly enhanced (Duchek, 2018). This would affect their negative tendencies toward challenging sustainable entrepreneurship. Conversely, when individuals with dark triad have a low level of psychological resilience, their negative attitude toward sustainable entrepreneurship may persist.

In a sense, psychological resilience can motivate individuals to pursue long-term interests and focus on solving the challenges of environmental and social issues, and positively affect their SEO. Considering the relationship between resilience and coping ability (Linnenluecke, 2017), and the key role of resilience in entrepreneurship, we believe that resilience may help reduce the negative impact of the dark triad on SEO. That is to say, for individuals with the dark triad, the higher their psychological resilience, the lower the negative impact of the dark triad traits on SEO, and vice versa.

**H3: Psychological resilience moderates the relationship of (a) Machiavellianism, (b) psychopathy, and (c) Narcissism with SEO.**

## Materials and Methods

### Participants

According to Hmieleski and Lerner (2016), a sample of business management students is appropriate to study the dark triad and entrepreneurship, as they generally represent business-oriented individuals and are unlikely to show omitted variable bias and endogeneity. Therefore, a sample of MBA students of Tianjin University was used in this study.

### Ethics Statement

Ethics approval for this research was not required as per institutional and national guidelines. Consent from all research participants was obtained by virtue of survey completion.

### Data Collection and Procedure

Measurements of the dark triad, psychological resilience, and SEO are well-developed and validated. According to the published scale in English, we translated all the items from English to Chinese and then translated them into English to ensure that they were used correctly and appropriate for the Chinese context. In order to minimize the risk of common method variance (CMV), we have done corresponding work in research design and data collection. At the stage of questionnaire design, we randomly arranged the order of all items and reduced participants’ guesses about the purpose of measurement (Podsakoff et al., 2003). At the same time, in the course of the survey, we told the participants that all the data were used for academic research and were anonymous and confidential (Podsakoff et al., 2003). Before conducting the formal survey, we randomly selected 20 volunteers to pilot the questionnaire. Based on their feedback and considering language expression habits in the Chinese context, we established the final questionnaire. All items in the questionnaire were rated on a seven-point Likert scale, ranging from 1 (“highly disagree”) to 7 (“highly agree”).

We obtained a full list of MBA students from the MBA Education and Management Center of Tianjin University, totaling 600 students. Subsequently, we selected 420 students by stratified sampling according to grade. Next, trained investigators distributed questionnaires to the students in their free time and collected the completed questionnaires on the spot to ensure the quality of the answers. Data collection was mainly concentrated in the period from May 15 to June 5, 2018. Finally, we collected a total of 402 questionnaires (95.71% response rate). After eliminating 74 questionnaires that were incomplete or invalid, the final sample consisted of 328 valid questionnaires, which implies an effective sampling rate of 81.59%.

### Measures

#### The Dark Triad

The Dark Triad Dirty Dozen scale, developed by Jonason and Webster (2010), has been widely used and validated to assess the dark triad. This study used this scale to separately measure the three dark triad traits. The scale consists of 12 items, with each dark triad trait being measured with four items. Items measuring narcissism include “I tend to seek prestige or status,” “I tend to want others to admire me,” and so on. Psychopathy items include “I tend to lack remorse,” “I tend to be callous or insensitive,” and so on. Items assessing Machiavellianism include “I have used deceit or lied to get my way,” “I tend to exploit others toward my own end,” and so on. We assessed narcissism, psychopathy, and Machiavellianism, respectively, calculating the average of the four items as the final score of each personality trait. The higher the score, the higher the corresponding personality level.

#### Psychological Resilience

To measure resilience, we used the scale of Manzano-García and Ayala Calvo (2013), who developed a Spanish adaptation of the Connor–Davidson Resilience Scale (CD-RISC). The instrument was developed using a sample of entrepreneurs and has very good psychometric properties (Manzano-García and Ayala Calvo, 2013). The revised instrument measures psychological resilience with 23 items, such as “I can deal with whatever comes my way,” “I have a strong sense of purpose,” and “I can handle unpleasant feelings.”

#### Sustainable Entrepreneurial Orientation

In this paper, the measurement of SEO was mainly based on the research of Kuckertz and Wagner (2010), in which SEO was assessed with five items evaluating sustainability and environmental attitudes: “Firms should take an internationally leading role in the field of environmental protection,” “The environmental performance of a company will be considered more and more by financial institutions in the future,” “I think that environmental problems are one of the biggest challenges for our society,” “I think that entrepreneurs and companies need to take on a larger social responsibility,” and “Firms that are environmentally oriented have advantages in recruiting and retaining qualified employees.”

#### Control Variables

According to the existing literature on entrepreneurial orientation, the control variables selected in this study included age, gender, marital status, and family business situation. Older students often have more work experience and wider social connections, which may make them more inclined to consider starting their own businesses (Kautonen et al., 2015). Studies have shown that gender is associated with individual entrepreneurial orientation, and male entrepreneurial orientation is higher (Goktan and Gupta, 2015). In view of the fact that entrepreneurship often involves high risks, married individuals’ SEO may be relatively low. Considering that the impact of the family environment is usually significant and far-reaching, individuals with family businesses may attach greater importance to long-term interests and be more likely to engage in sustainable entrepreneurship.

### Reliability and Validity

We tested the reliability and validity of the scale in SPSS 22.0. The reliability of the study questionnaire were tested using Cronbach’s alpha and composite reliabilities (CR). In the reliability analysis, Cronbach’s alpha for all constructs was greater than 0.70 (Table 1), which indicates good internal consistency (Cronbach, 1951). Moreover, as shown in Table 1, the CR values of all constructs were higher than the recommended minimum standard of 0.70 (Hair et al., 2010), so the measures in this study had good internal consistency.

**TABLE 1:** Measurement items and reliabilities.

**Variables**	**Items**	**Factor loading**	**Alpha**	**AVE**	**CR**
Narcissism	I tend to want others to admire me.	0.710	0.795	0.625	0.869
	I tend to seek prestige or status.	0.731			
	I tend to expect special favors from others.	0.855			
	I tend to want others to pay attention to me.	0.854			
Psychopathy	I tend to lack remorse.	0.803	0.773	0.604	0.856
	I tend to be unconcerned with the morality of my actions.	0.846			
	I tend to be callous or insensitive.	0.855			
	I tend to be cynical.	0.569			
Machiavellianism	I tend to manipulate others to get my way.	0.676	0.798	0.627	0.870
	I have used deceit or lied to get my way.	0.828			
	I have used flattery to get my way.	0.834			
	I tend to exploit others toward my own end.	0.818			
Psychological resilience	I am able to adapt to change.	0.739	0.952	0.526	0.962
	I have close and secure relationships.	0.688			
	I can deal with whatever comes my way.	0.652			
	Past success gives me confidence for new challenges.	0.716			
	I see the humorous side of things.	0.731			
	Coping with stress strengthens me.	0.823			
	I tend to bounce back after a hardship or illness.	0.762			
	I give my best effort, no matter what.	0.736			
	I can achieve my goals.	0.798			
	When things look hopeless, I don’t give up.	0.674			
	I know where to turn to for help.	0.752			
	Under pressure, I focus and think clearly.	0.817			
	I prefer to take the lead in problem solving.	0.760			
	I am not easily discouraged by failure.	0.824			
	I think of myself as a strong person	0.779			
	I can make unpopular or difficult decisions.	0.593			
	I can handle unpleasant feelings.	0.708			
	I have to act on a hunch.	0.547			
	I have a strong sense of purpose.	0.671			
	I feel in control of my life.	0.703			
	I like challenges.	0.746			
	I work to attain my goals.	0.792			
	I take pride in my achievements.	0.576			
Sustainable entrepreneurial orientation	Firms should take an internationally leading role in the field of environmental protection.	0.817	0.870	0.661	0.907
	The environmental performance of a company will in future be considered more and more by financial institutions.	0.829			
	I think that environmental problems are one of the biggest challenges for our society.	0.799			
	I think that entrepreneurs and companies need to take on a larger social responsibility.	0.854			
	Firms that are environmentally oriented have advantages in recruiting and retaining qualified employees.	0.763			

*This study measures all items with a seven-point Likert scale.*

Furthermore, we tested the validity of the scale by factor loading, average variance extracted (AVE), and the square root of the AVE. These results indicate that the theoretical structure of this study had good psychometric characteristics. We used confirmatory factor analysis to test the convergent validity of the scale. Previous studies have shown that factor loadings greater than 0.70 indicate that about half of the variance of items can be attributed to the construct, which is a marker of construct validity (Götz et al., 2010). In this study, the factor loadings of all items were between 0.547 and 0.855 (Table 1), which fully demonstrates a close relationship between items and constructs and that the questionnaire conforms to the requirements of convergent validity. Moreover, the AVE scores of all constructs in this study were higher than the recommended minimum value of 0.50 (Table 1). This further indicates that the scale meets the requirement of convergent validity. In addition, we used the square root of the AVE to test the discriminant validity of the scale. The diagonal elements in Table 2 are the square root of the AVE for each construct, which were obviously larger than the non-diagonal elements. This indicates that the scale as a whole meets the criteria for discriminant validity (Fornell and Larcker, 1981). In conclusion, we believe that the reliability and validity of this study questionnaire can meet the requirements of further research.

**TABLE 2:** Mean, standard deviation, and correlation of study variable^a^.

	**Mean**	***SD***	**1**	**2**	**3**	**4**	**5**	**6**	**7**	**8**	**9**
(1) Sex	1.520	0.500	N/A								
(2) Age	31.060	4.099	-0.212^∗∗^	N/A							
(3) Married	1.450	0.499	0.218^∗∗^	-0.480^∗∗^	N/A						
(4) Family business	1.730	0.445	0.002	-0.168^∗∗^	0.061	N/A					
(5) Narcissism	3.116	1.371	-0.124^∗^	-0.057	-0.003	-0.060	**0.791**				
(6) Psychopathy	2.881	1.346	-0.105	-0.136^∗^	0.064	-0.016	0.738^∗∗^	**0.777**			
(7) Machiavellianism	4.463	1.259	-0.179^∗∗^	-0.039	-0.046	-0.071	0.521^∗∗^	0.390^∗∗^	**0.792**		
(8) Psychological resilience	5.356	0.863	-0.165^∗∗^	-0.202^∗∗^	-0.145^∗∗^	-0.181^∗∗^	0.019	-0.158^∗∗^	0.038	**0.725**	
(9) SEO	5.598	1.044	-0.091	0.143^∗^	-0.053	-0.136^∗^	-0.174^∗∗^	-0.257^∗∗^	0.130^∗^	0.483^∗∗^	**0.813**

^a^*N* = 328. *Correlation is significant at the 0.05 level (two-tailed). **Correlation is significant at the 0.01 level (two-tailed). *(a)* N/A refers to this item is not adaptive to analysis and *(b)* The diagonal elements in bold are square roots of AVE.

## Results

**FIGURE 1:**
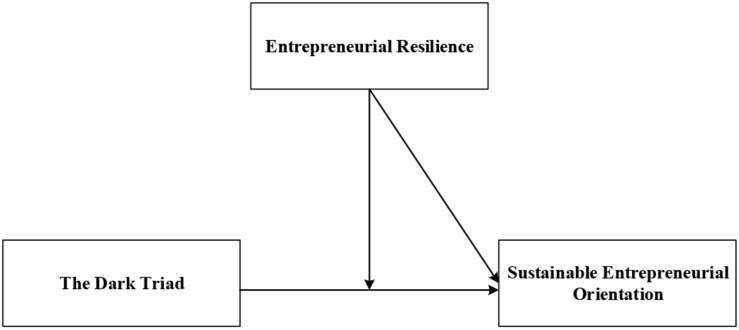
This study’s research model.

Based on social exchange theory and social cognition theory, this study explored the effects of the dark triad traits and psychological resilience on SEO, and further analyzed the moderating effect of psychological resilience on the relationship between the dark triad and SEO. Figure 1 illustrates the theoretical model.

Table 2 presents descriptive statistics and correlations of variables. There was a significant correlation between the key variables. At the same time, the correlation coefficients between variables were less than 0.75, which ruled out multiple collinearity to some extent.

Each hypothesis was verified using hierarchical regression analysis. Beforehand, we ensured that our data were consistent with the assumptions of regression analysis. We used the variance inflation factor (VIF) to examine the possibility of multicollinearity. The results showed that the maximum VIF value was 2.57, much lower than the cut-off value of 10 proposed by Kleinbaum et al. (1998), which indicated no multicollinearity in the regression model. Furthermore, in all models, the Durbin–Watson statistic showed that the sequence correlation of residuals was within the acceptable range (1.50 and 2.50) (Ayala and Manzano, 2014), confirming no auto-correlation problems in the data.

Table 3 shows the hierarchical regression results for main, interaction, and control effects. First, only age (*p* < 0.05) was significant factors of SEO when all control variables were included in Model 1. Second, we added the dark triad to Model 2 as independent variables and kept SEO as the dependent variable. The results showed that Machiavellianism had a slightly negative correlation with SEO (β = −0.116, *p* < 0.10) and psychopathy was negatively associated with SEO (β = −0.193, *p* < 0.01), but narcissism was positively correlated with SEO (β = −0.239, *p* < 0.001). Therefore, (H1a, H1b) were supported, but (H1c) was not supported. Third, Model 3 included psychological resilience as the independent variable and SEO as the dependent variable. The results showed that psychological resilience had a significant positive effect on SEO (β = 0.563, *p* < 0.001); thus, H2 was verified.

**TABLE 3:** Results of the hierarchical regression analysis.

	**Sustainable entrepreneurial orientation**								
	**Model 1**	**Model 2**	**Model 3**	**Model 4 main effects**	**Model 5 interaction**	**Model 6 main effects**	**Model 7 interaction**	**Model 8 main effects**	**Model 9 interaction**
Sex	-0.146	-0.141	-0.017	-0.070	-0.026	-0.077	-0.028	0.030	0.028
Age	0.034^∗^	0.029+	0.018	0.013	0.015	0.012	0.016	0.022	0.018
Married	0.104	0.137	0.138	0.133	0.097	0.144	0.117	0.149	0.137
Family business	-0.251+	-0.227+	-0.083	-0.115	-0.111	-0.099	-0.093	-0.059	-0.061
Machiavellianism		-0.116+		-0.144^∗∗∗^	-0.150^∗∗∗^				
Psychopathy		-0.193^∗∗^				-0.145^∗∗∗^	-0.0151^∗∗∗^		
Narcissism		0.239^∗∗∗^						0.096^∗^	0.108^∗^
Psychological resilience			0.563^∗∗∗^	0.563^∗∗∗^	0.495^∗∗∗^	0.527^∗∗∗^	0.443^∗∗∗^	0.564^∗∗∗^	0.591^∗∗∗^
Machiavellianism* Psychological resilience					0.167^∗∗∗^				
Psychopathy* Psychological resilience							0.191^∗∗∗^		
Narcissism * Psychological resilience									0.076+
*R*^2^	0.037	0.159	0.237	0.272	0.328	0.271	0.336	0.250	0.257
Δ*R*^2^	0.025^∗^	0.140^∗∗∗^	0.225^∗∗∗^	0.258^∗∗∗^	0.313^∗∗∗^	0.257^∗∗∗^	0.321^∗∗∗^	0.236^∗∗∗^	0.240^∗∗∗^
Durbin–Watson	1.817	1.905	1.773	1.859	1.902	1.879	1.887	1.708	1.711

*N* = 328. ^+^*p* < 0.10; *p < 0.05; **p < 0.01; ^∗∗∗^*p* < 0.001.

Finally, in order to test H3, we standardized the independent variables before we created each interactive item (Cohen et al., 2003). Model 4 took Machiavellianism and psychological resilience as independent variables. On this basis, Model 5 introduced an interactive item between Machiavellianism and psychological resilience, and psychological resilience was found to moderate the relationship between Machiavellianism and SEO (β = 0.167, *p* < 0.001), weakening the negative impact of Machiavellianism on SEO. Model 6 took psychopathy and psychological resilience as independent variables. On this basis, Model 7 introduced an interactive item between psychopathy and psychological resilience. The results showed that psychological resilience moderated the relationship between psychopathy and SEO (β = 0.191, *p* < 0.001), weakening the negative impact of psychopathy on SEO. Model 8 took narcissism and psychological resilience as independent variables. On this basis, Model 9 introduced an interactive item between narcissism and psychological resilience. The results showed a moderating effect of psychological resilience on the relationship between narcissism and SEO (β = −0.076, *p* < 0.10), with psychological resilience marginally weakening the positive effect of narcissism on SEO. Therefore, (H3a, H3b, and H3c) were supported.

**FIGURE 2:**
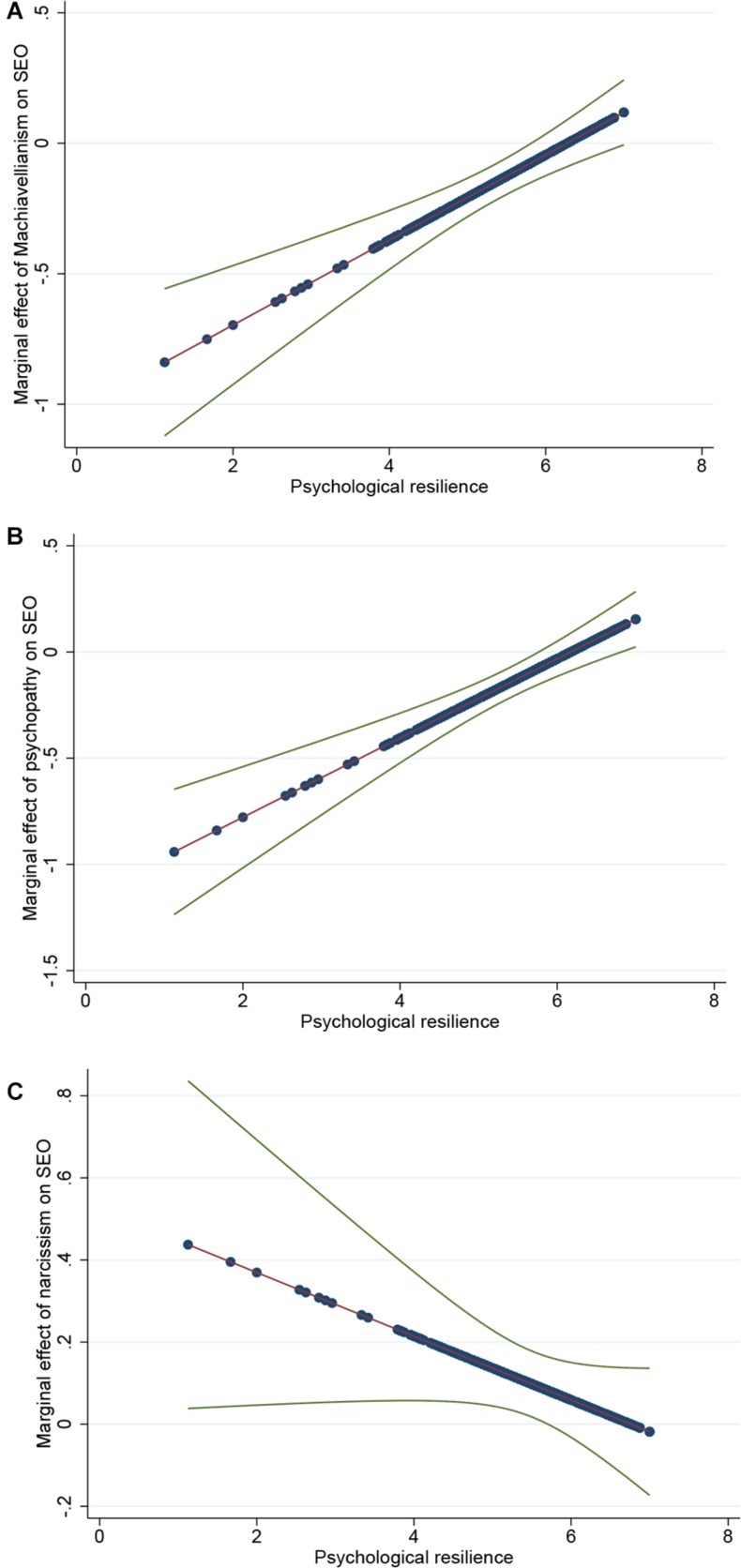
Marginal effect of the dark triad (Machiavellianism, psychopathy and narcissism) on SEO for the whole range of psychological resilience. **(A)** Marginal effect of Machiavellianism on SEO. **(B)** Marginal effect of psychopathy on SEO. **(C)** Marginal effect of narcissism on SEO.

To further analyze the moderating effect of psychological resilience, we plotted the marginal effect for the whole range of psychological resilience (see Figure 2). Our drawing procedure followed the work of Meyer et al. (2017). In Figure 2, the area between the two outer lines is the 95% confidence interval around the interaction line. Moreover, when the two lines reflecting the confidence interval are both below and above the horizontal zero line, the interaction effect is significant (Meyer et al., 2017). Figure 2A shows the impact of Machiavellianism on SEO for all levels of psychological resilience. As Figure 2A notes, Machiavellianism had a significant negative effect on SEO when psychological resilience was low, but no significant effect when psychological resilience was high. Figure 2B shows the impact of psychopathy on SEO for all levels of psychological resilience. As Figure 2B indicates, when psychological resilience was low, psychopathy had a negative impact on SEO; when psychological resilience was high, psychopathy had no significant impact on SEO. Figure 2C shows the impact of narcissism on SEO for all levels of psychological resilience. As Figure 2C shows, narcissism had a positive effect on SEO when psychological resilience is low, but no significant effect when psychological resilience was high.

## Discussion

Our study examined the relationship between the three elements of the dark triad, psychological resilience, and individual SEO in a sample of MBA students in Tianjin University in China. Not only did we analyze the influence of the dark triad traits and psychological resilience on SEO, but also explored the moderating effect of psychological resilience on the relationship between the dark triad and SEO.

First, we found differences in the influence of the three elements of the dark triad on SEO. Machiavellianism and psychopathy had a negative impact on SEO, which is consistent with our hypotheses. Individuals with Machiavellianism always try to seek immediate gratification by adopting short-term strategies that are closely related to living a “fast life” (Jonason and Webster, 2010), and long-term environmental benefits are not important to them. Moreover, individuals with Machiavellianism are mostly self-interested and pay less attention to the consequences for those around them and their surroundings (Zettler et al., 2011). As for individuals with psychopathy, they simply do not care about the consequences of their actions on the environment or society, and seriously lack social responsibility (Boddy, 2015). Most importantly, individuals high in psychopathy often lack empathy, and may be inclined to turn a blind eye to increasingly serious environmental problems and even do things that are detrimental to the environment. However, we were very surprised to find that narcissism had a significantly positive impact on SEO, which is inconsistent with our original expectations. We think this may be because narcissism is often two-sided (Myung and Yun, 2017), that is, narcissism has both a negative side and a positive side. In the Chinese context, narcissists may have a more optimistic attitude toward SEO, being inclined to integrate the concept of sustainable development into entrepreneurship.

Second, our study showed that psychological resilience was positively correlated with SEO, which is consistent with our original hypothesis. We believe that individuals with high psychological resilience are usually more successful in coping with major changes, adversities, or risks (Duchek, 2018), and thus have the courage and determination to achieve long-term benefits. Moreover, the three characteristics of psychological resilience (i.e., hardiness, resourcefulness, and optimism) have a positive role in promoting sustainable entrepreneurship among potential entrepreneurs. In conclusion, individuals with high psychological resilience can often take positive actions in adversity. They have higher motivation and more positive attitude to solve environmental and social problems around them, and can adhere to the concept of sustainable development more firmly, thereby achieving long-term environmental and other benefits.

Third, the moderating effect of psychological resilience on the relationship between the dark triad and SEO was verified. Psychological resilience weakened the negative impact of both Machiavellianism and psychopathy on SEO. In other words, when individuals have only Machiavellianism/psychopathy, they will usually be reluctant to start sustainable businesses. However, if they also have psychological resilience, their willingness to oppose sustainable entrepreneurship will be weakened, and they may even have a certain tendency toward SEO.

## Conclusion

### Research Implications

The current study makes important contributions to research in this area. First, our study simultaneously analyzed the influence of bright and dark personality traits on SEO. Existing studies only focused on the impact of one aspect of personality (i.e., bright- or dark-side personality traits) on entrepreneurial orientation. Considering that individual personality is multifaceted, our study introduced both the bright and the dark side of personality. We selected psychological resilience and the dark triad as pre-variables, as they are particularly critical in the entrepreneurial environment, and analyzed their impact on SEO from a comprehensive perspective, which makes a valuable contribution to research on personality and entrepreneurial orientation.

Second, by separately analyzing the influence of the dark triad and psychological resilience on SEO, we also assessed the moderating effect of psychological resilience on the relationship between the dark triad and SEO, which responds to the appeal of Smith et al. (2017) to attend to the interaction of dark and bright personality traits when studying personality factors in entrepreneurial situations. Moreover, considering that the interaction between personality traits may also play an important role in SEO, we analyzed the moderating effect of psychological resilience and explored the antecedent factors of SEO from a new perspective.

Third, our study focused on the influence of personality on SEO at the individual level. Previous studies focused on the impact of personality on organizational entrepreneurial orientation (Wales et al., 2013; Engelen et al., 2015). Considering that research in the field of entrepreneurship is increasingly focused on the critical role of individuals, we adopted an individual perspective. At the same time, the purpose of entrepreneurship is not only to obtain economic value, but social value and environmental benefits are increasingly becoming the focus of business. Therefore, our study focused on the impact of personality traits on individual SEO, laying the foundation for further analysis of the factors affecting sustainable entrepreneurship at the individual level.

### Implications for Practice

Our work has two important implications for China’s entrepreneurship education. First, when conducting entrepreneurship education, business school teachers should pay attention to the dark personality traits of students and moderately intervene and guide them. Our results showed that psychopathy and Machiavellianism were negatively correlated with SEO. As we have seen, students with psychopathy and Machiavellianism may have a certain negative perception of society and pay more attention to short-term interests and fast-life strategies. This is very unfavorable to the current entrepreneurial environment and its development in the future. Therefore, business school teachers should take appropriate measures and implement educational interventions to reduce students’ level of psychopathy and Machiavellianism with specific teaching practices, thereby reducing their resistance to SEO.

Second, business school teachers should teach students in accordance with their aptitude on the basis of their personality characteristics. Especially for students with psychological resilience, business school teachers should formulate targeted entrepreneurial education programs according to their personality traits, and give full play to the advantages of students’ psychological resilience in entrepreneurship and innovation. Moreover, our study found that psychological resilience contributed to reduce the negative impact of psychopathy and Machiavellianism on SEO. Therefore, we suggest that business school teachers should use the necessary methods to build psychological resilience among students who lack it Bullough and Renko (2013). For example, strengthening students’ cultivation of psychological resilience may reduce the negative effects of psychopathy and Machiavellianism as much as possible. In addition, in order to cultivate students’ concept of sustainable development more effectively, it is necessary for business school teachers to incorporate sustainable concepts into their educational practice and influence students’ SEO in subtle ways, for example, by adding sustainable ideas to the curriculum design. Although this is a minor influence, it is especially important.

### Limitations and Future Directions

First, the conclusions of this study are based on data collected in the Chinese context, which may be different from other countries. This study found that psychopathy and Machiavellianism had a negative impact on SEO, while narcissism had a positive impact on SEO. However, in other countries, the results may differ. Specific social issues and serious environmental problems in China’s current development may affect the entrepreneurial attitude of potential entrepreneurs to a certain extent (Huang et al., 2018). Therefore, future research should consider collecting data from different countries in order to understand the differences in the impact of individual personality traits on SEO (Chang and Park, 2018).

Second, self-reported questionnaires may lead to a certain degree of CMV (Do and Dadvari, 2017). This study controlled for this by adjusting the order of questions and reducing participants’ guesswork about the purpose of measurement; moreover, the low-risk setting of this study was sufficient to rule out forgery (i.e., participants had no motive to falsify their responses) (Akhtar et al., 2013). However, in order to control the effect of CMV more strictly, future research may consider having participants answer the same questions at different time points or adopting different methods to measure personality characteristics more objectively and comprehensively, and thus provide more powerful support for the research hypotheses.

Third, our study lacked a detailed classification of narcissism. Our study only analyses narcissism in the dark triad, which is indeed a limitation. Because different types of narcissism have different characteristics, we need to further analyze how different types of narcissism affect SEO in future research. Considering that narcissism is generally divided into grandiose and vulnerable narcissism (Wink, 1991; Miller et al., 2011), future research should focus on narcissism in subdivision studies and comparative studies to further reveal the influence mechanism of distinct types of narcissism on SEO.

## Ethics Statement

Ethics approval for this research was not required as per institutional and national guidelines. Consent from all research participants was obtained by virtue of survey completion.

## Author Contributions

All authors listed have made a substantial, direct and intellectual contribution to the work, and approved it for publication.

## Conflict of Interest Statement

The authors declare that the research was conducted in the absence of any commercial or financial relationships that could be construed as a potential conflict of interest. The reviewer LC declared a past co-authorship with one of the authors FG to the handling Editor.
